# Fabrication and NO_2_ gas sensing performance of TeO_2_-core/CuO-shell heterostructure nanorod sensors

**DOI:** 10.1186/1556-276X-9-638

**Published:** 2014-11-27

**Authors:** Sunghoon Park, Soohyun Kim, Gun-Joo Sun, Wan In Lee, Kyoung Kook Kim, Chongmu Lee

**Affiliations:** 1 Department of Materials Science and Engineering, Inha University, 253 Yonghyun-dong, Nam-gu, Incheon 402-751, Republic of Korea; 2 Department of Chemistry, Inha University, 253 Yonghyun-dong, Nam-gu, Incheon 402-751, Republic of Korea; 3 Department of Nanooptical Engineering, Korea Polytechnic University, 2121 Jeongwangdong, Shiheung-city, Gyeonggido 429-793, Republic of Korea

**Keywords:** TeO_2_ nanorods, CuO shells, Gas sensors, Response, NO_2_

## Abstract

TeO_2_-nanostructured sensors are seldom reported compared to other metal oxide semiconductor materials such as ZnO, In_2_O_3_, TiO_2_, Ga_2_O_3_, etc. TeO_2_/CuO core-shell nanorods were fabricated by thermal evaporation of Te powder followed by sputter deposition of CuO. Scanning electron microscopy and X-ray diffraction showed that each nanorod consisted of a single crystal TeO_2_ core and a polycrystalline CuO shell with a thickness of approximately 7 nm. The TeO_2_/CuO core-shell one-dimensional (1D) nanostructures exhibited a bamboo leaf-like morphology. The core-shell nanorods were 100 to 300 nm in diameter and up to 30 μm in length. The multiple networked TeO_2_/CuO core-shell nanorod sensor showed responses of 142% to 425% to 0.5- to 10-ppm NO_2_ at 150°C. These responses were stronger than or comparable to those of many other metal oxide nanostructures, suggesting that TeO_2_ is also a promising sensor material. The responses of the core-shell nanorods were 1.2 to 2.1 times higher than those of pristine TeO_2_ nanorods over the same NO_2_ concentration range. The underlying mechanism for the enhanced NO_2_ sensing properties of the core-shell nanorod sensor can be explained by the potential barrier-controlled carrier transport mechanism.

**PACS:**

61.46. + w; 07.07.Df; 73.22.-f

## Background

In recent years, one-dimensional (1D) nanostructure-based sensors attracted considerable attention owing to their high surface-to-volume ratios [1-5]. Considerable effort has been made to develop 1D nanostructured gas sensors with good sensing performances, but further improvements in the sensitivity of 1D nanostructured sensors are needed. The fabrication of heterostructures [6-8] is a promising technique to improve the sensitivity of the 1D nanostructured sensors. The improved sensing performance of the heterostructured 1D sensors has been attributed to a range of factors including increased potential barriers at the interface of the heterostructure [9,10], modulated depletion layer [11,12], band bending due to equilibration of the Fermi energy levels [13], synergistic surface reactions [14], etc.

Paratellurite (α-TeO_2_) is a metal oxide semiconductor with a distorted rutile structure [15]. TeO_2_ has applications in optical storage, laser devices and gas sensors, dosimeters, modulators, and deflectors owing to its unique properties such as high refractive index and high optical nonlinearity [16]. TeO_2_-nanostructured sensors have attracted less attention compared to other metal oxide semiconductor materials such as ZnO, In_2_O_3_, TiO_2_, Ga_2_O_3_, etc. In 2007, Liu et al. [17] synthesized TeO_2_ nanowires that were sensitive to NO_2_, NH_3_, and H_2_S gases. According to their results, TeO_2_ 1D nanostructures are promising for producing low power consumption gas sensors. The incorporation of a surface decoration or heterostructure formation technique can improve their sensing performance further. In this regard, a recent study reported the sensing properties of Pt-doped TeO_2_ nanorods [16]. On the other hand, this paper reports the synthesis of TeO_2_-core/CuO-shell nanorods and the sensing properties of multiple networked TeO_2_-core/CuO-shell nanorod gas sensors toward NO_2_ gas. The underlying mechanism for the enhanced sensing performance of the core-shell nanorod sensors is also discussed.

## Methods

TeO_2_/CuO core-shell nanorods were synthesized using a two-step process: thermal evaporation of Te powder followed by sputter deposition of CuO. TeO_2_ nanorods were synthesized on a p-type Si (100) substrate in a quartz tube furnace by thermal evaporation of Te powder at 400°C in air without a metal catalyst or the supply of other gas. The thermal evaporation process was conducted at room temperature for 1 h and the furnace was cooled to room temperature. Subsequently, the TeO_2_ nanorods were coated with a thin CuO layer by sputtering a CuO target by radio frequency (RF) magnetron sputtering from a CuO target. The base and working pressure was 5.0 × 10^-6^ Torr and 2.0 × 10^-2^ Torr, respectively, and the N_2_ gas flow rate was 20 cm^3^/min throughout the evaporation process. The RF sputtering power and sputtering time were 100 W and 20 min, respectively.

The structure and morphology of the nanorod samples were characterized by scanning electron microscopy (SEM, Hitachi S-4200, Billerica, MA, USA), transmission electron microscopy (TEM, Philips CM-200, Eindhoven, the Netherlands), and selected area electron diffraction. X-ray diffraction (XRD, Philips X’pert MRD, Eindhoven, the Netherlands) patterns were performed using Cu K_α_ radiation (0.15406 nm). Energy-dispersive X-ray spectroscopy (EDS) was carried out to examine the elemental composition of the core-shell nanorod samples. The resistance of multiple networked pristine TeO_2_ nanorod and TeO_2_/CuO core-shell nanorod sensors were measured using a Keithley source meter-2612 at a source voltage of 10 V at 150°C and 50% RH. The 50% relative humidity might be somewhat high for sensing tests. A flow-through technique was used to test the gas sensing properties. NO_2_ gas diluted with synthetic air at different ratios was injected into the testing tube at a constant flow rate of 200 cm^3^/min. The detailed procedures for sensor fabrication and the sensing test are reported elsewhere [18].

## Results and discussion

**Figure 1:**
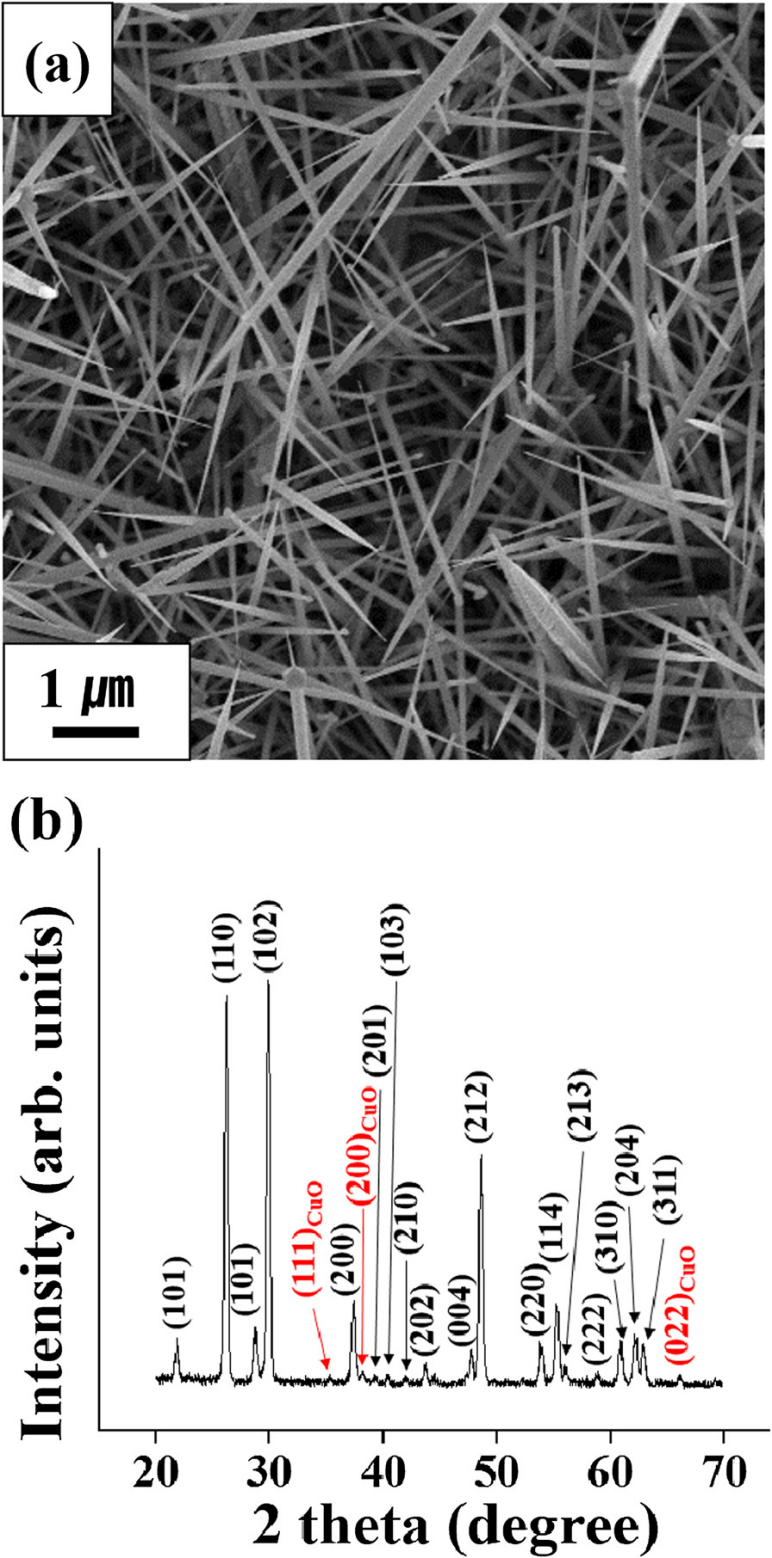
**SEM image (a) and XRD patterns (b) of TeO**_**2**_**/CuO core-shell nanorods.**

Figure 1a shows a SEM image of the TeO_2_/CuO core-shell nanorods prepared by thermal evaporation followed by sputtering. Each 1D nanostructure exhibited a rod-like morphology with a sharp tip, i.e. a bamboo leaf-like morphology. The core-shell nanorods were 100 to 300 nm in diameter and up to 30 μm in length. XRD was performed to determine the crystal structures of the core-shell nanorods. The XRD patterns of the TeO_2_/CuO core-shell nanorods showed that the TeO_2_ cores were crystalline, whereas the CuO shells were polycrystalline (Figure 1b). Most of the XRD peaks of the TeO_2_/CuO core-shell nanorods were assigned to be the reflections of primitive tetragonal-structured rutile-type TeO_2_. In addition, three small reflection peaks were assigned to the 111, 200, and 022 reflections of monoclinic-structured CuO with lattice constants of *a* = 0.4689 nm, b = 0.342 nm, c = 0.513 nm, and β = 99.57° (JCPDS No. 89–5899).

**Figure 2:**
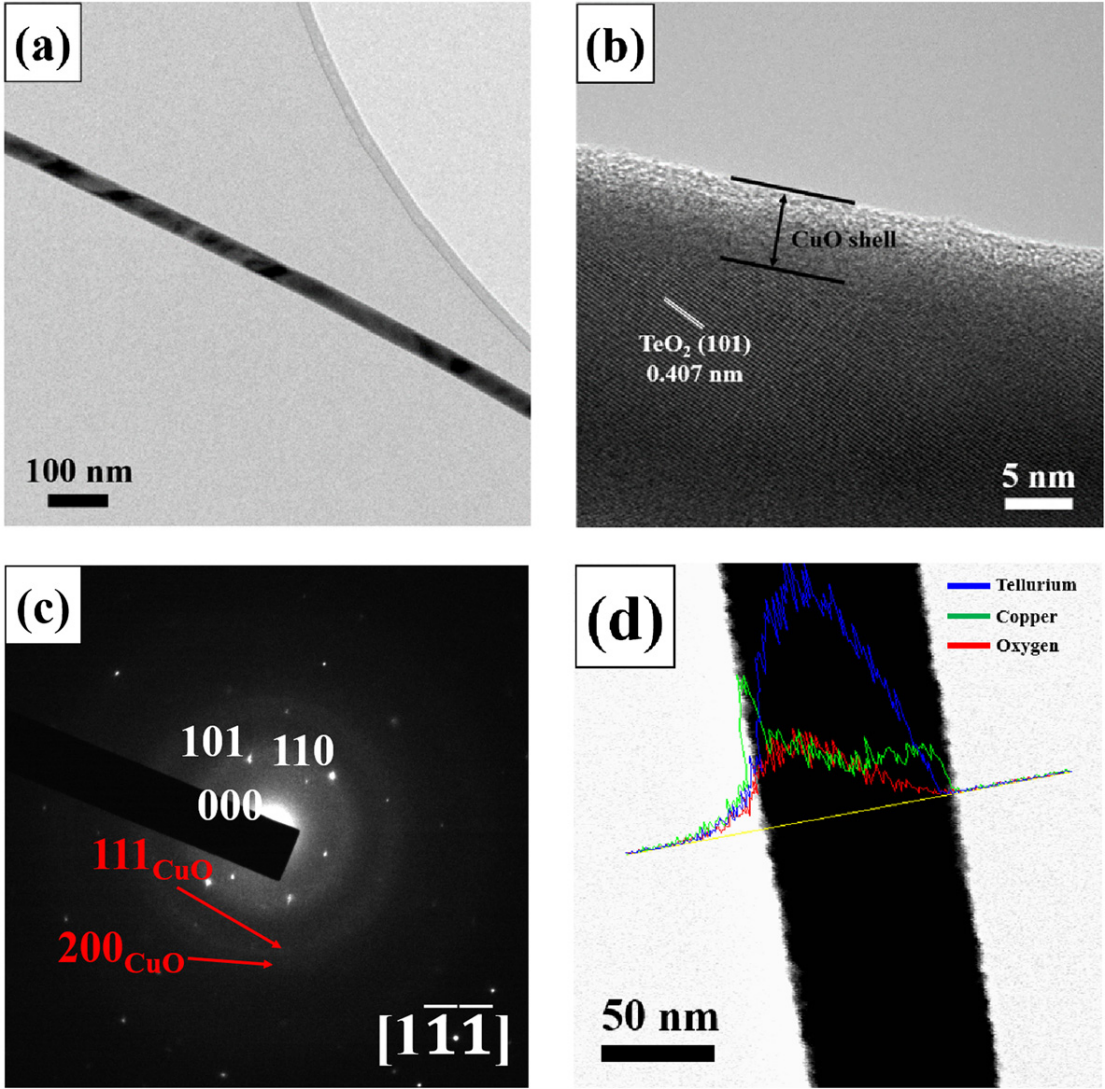
**TEM images, diffraction pattern, and profile of TeO**_**2**_**/CuO core-shell nanorods. (a)** Low-magnification TEM image, **(b)** high-resolution TEM image, **(c)** selected area electron diffraction pattern, and **(d)** EDS line scanning concentration profile of TeO_2_/CuO core-shell nanorods.

The low-magnification TEM image of a typical core-shell nanorod showed that the nanorod had a uniform diameter along its length direction (Figure 2a). TEM revealed a shell width of approximately 7 nm. A close examination of the high-resolution TEM (HRTEM) image (Figure 2b) shows a fringe pattern in the core region (the lower darker region), suggesting it to be a single crystal. The clear spots in the corresponding selected area electron diffraction (SAED) pattern were assigned to the primitive tetragonal structured TeO_2_ with lattice constants of *a* = 0.4810 nm and *c* = 0.7613 (JCPDS No. 78–1713) (Figure 2c). On the other hand, the halo-like concentric ring pattern might be due to the polycrystalline CuO shell. The line-scanning EDS concentration profile along the diameter of a typical core-shell nanorod (Figure 2d) revealed a higher Te concentration in the center region and a higher Cu concentration in both edge regions of the nanorod, confirming the TeO_2_-core/CuO-shell structure.

**Figure 3:**
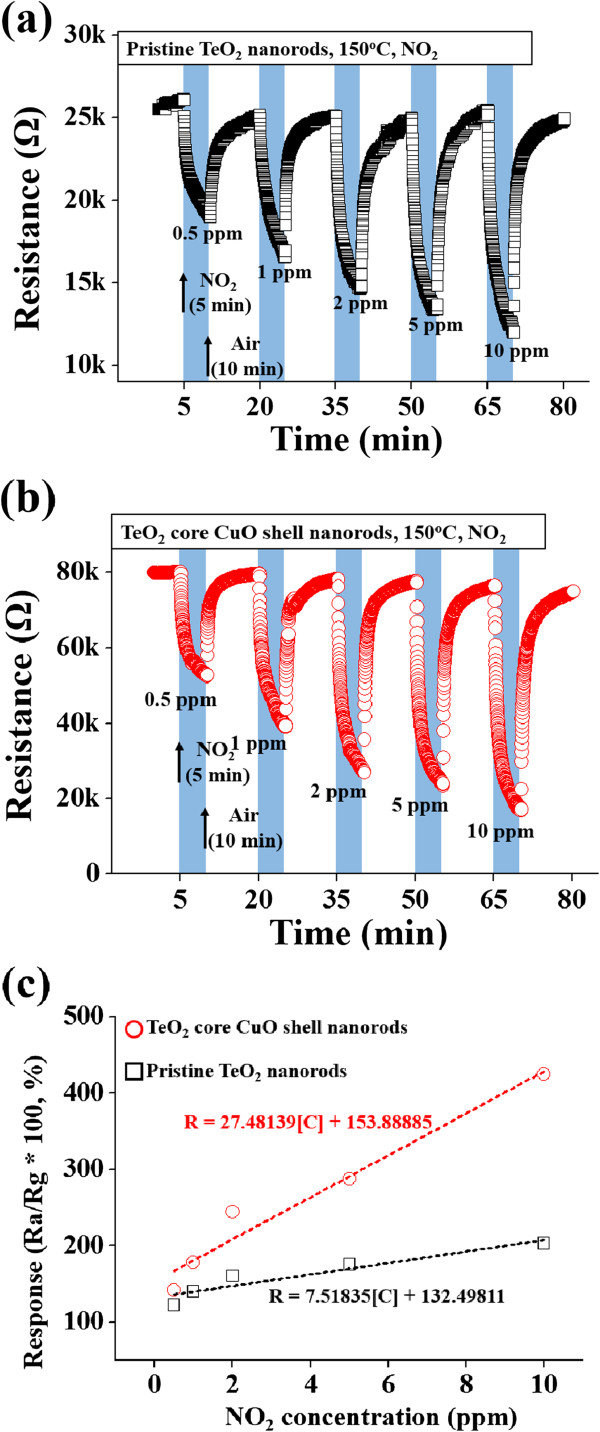
**Responses of the pristine TeO**_**2 **_**nanorod and TeO**_**2**_**/CuO core-shell nanorod gas sensors.** Dynamic responses of **(a)** the pristine TeO_2_ nanorod and **(b)** TeO_2_/CuO core-shell nanorod gas sensors to NO_2_ at 150°C. **(c)** Responses of the pristine TeO_2_ nanorod and core-shell nanorod gas sensors as a function of NO_2_ gas concentration.

Figure 3a,b shows the dynamic electrical responses of pristine TeO_2_ nanorods and TeO_2_/CuO core-shell nanorods, respectively, to NO_2_ at 150°C under 50% RH. The sensors were exposed to successive pulses of 0.5- to 10-ppm NO_2_ gas. The relative response of the p-type TeO_2_/CuO nanorod sensors is defined as *R*_*a*_*/R*_*g*_ for NO_2_, where *R*_*a*_ and *R*_*g*_ are the electrical resistances in the sensors in air and target gas, respectively. In all cases, the resistance returned to its original value after the NO_2_ gas flow was switched off, confirming the reversibility of the gas absorption and desorption processes. The pristine TeO_2_ nanorods showed responses of approximately 123% to 203% to NO_2_ at 0.5 to 10 ppm (Table 1). In contrast, the TeO_2_/CuO core-shell nanorods showed 1.2- to 2.1-fold stronger responses to NO_2_ than pristine TeO_2_ nanorod sensors at the same concentrations.

**Table 1:** **Responses of the TeO**_**2**_**/CuO nanorod sensor to NO**_**2 **_**gas at different concentrations at 150°C**

	**Response (R**_**a**_**/R**_**g**_**, %)**	
**NO**_**2 **_**concentration**	**Pristine TeO**_**2 **_**nanorod**	**TeO**_**2**_**/CuO nanorod**
0.5 ppm	122.60	142.17
1 ppm	140.27	178.73
2 ppm	160.08	244.24
5 ppm	175.51	287.80
10 ppm	203.12	424.91

Figure 3c compares the response to NO_2_ gas between pristine TeO_2_ nanorods and TeO_2_/CuO core-shell nanorods in the NO_2_ concentration range below 10 ppm. The response of an oxide semiconductor sensor can be expressed as R = A [C]^n^ + B, where A and B, n, and [C] are constants, exponent, and target gas concentration, respectively [19]. Data fitting gave R = 7.52 [C] + 132.5 and R = 27.48 [C] + 153.9 for the pristine TeO_2_ nanorod and TeO_2_-core/CuO-shell nanorod sensors, respectively. The core-shell nanorod sensor showed stronger response and higher increasing rate in response to NO_2_ gas at lower concentrations than the pristine nanorod sensor.

Table 2 lists the responses of the multiple networked pristine TeO_2_ nanorod sensor to NO_2_ gas along with those of other reported nanomaterial sensors. Overall, the sensing properties of the TeO_2_/CuO core-shell nanorod sensor fabricated in this study were comparable to those of other competing nanomaterials (Table 2), but the sensing test conditions such as operating temperature, gas concentration, etc. were different [20-31]. It should be noted that the NO_2_ concentration and the test temperature used in this study were mostly lower than those elsewhere. The responses of pristine TeO_2_ nanorods and TeO_2_-CuO nanorods to NO_2_ measured in this study were stronger than those of other metal oxides such as ZnO fibers, ZnO fibre mats, mesoporous WO_3_ thin film, and CdO nanowire measured at temperatures lower than 150°C. The response of WO_3_-doped SnO_2_ thin film was stronger to 500 ppm of NO_2_ than those of pristine TeO_2_ nanorods and TeO_2_-CuO nanorods to 10 ppm of NO_2_, but it should be noted that the former response was obtained to a far higher concentration of NO_2_. TiO_2_ nanofibers, SnO_2_ hollow spheres, and Ru-doped SnO_2_ nanowire showed stronger responses to NO_2_ than those of pristine TeO_2_ nanorods and TeO_2_-CuO nanorods, but their operation temperatures of the former were higher than 150°C. Pristine TeO_2_ nanorods and TeO_2_-CuO nanorods showed stronger responses than other metal oxide nanostructures except the above-mentioned nanomaterials.

**Table 2:** **Comparison of the responses of the TeO**_**2**_**/CuO core-shell nanorod sensor with those of other oxide 1D nanostructure sensors**

**Nanomaterial**	**Temperature (°C)**	**NO**_**2 **_**concentration (ppm)**	**Response (%)**	**Reference**
TeO_2_ nanorods	150	0.5	123	Present work
TeO_2_ nanorods	150	10	203	Present work
TeO_2_-CuO nanorods	150	0.5	142	Present work
TeO_2_-CuO nanorods	150	10	425	Present work
ZnO nanorods	300	0.1	35	[20]
ZnO nanowire	250	20	>95	[21]
ZnO nanobelt	350	8.5	81	[22]
ZnO fibers	100	0.4	50	[23]
WO3-core/ZnO-shell nanorods	300	5	281	[24]
TiO_2_ nanofibers	300	0.25	7,430	[25]
In-doped SnO_2_ nanoparticles	250	500	100	[26]
SnO_2_ nanoribbon	RT	3	116	[27]
SnO_2_ hollow spheres	160	5	1,150	[28]
Ru-doped SnO_2_ nanowire	150	200	>3,000	[29]
WO_3_-doped SnO_2_ thin film	100	500	2,210	[30]
In_2_O_3_ nanowires	400	50	360	[31]
In_2_O_3_ nanowires	250	50	200	[32]
WO_3_ nanorods	300	1	200	[33]
Au-doped WO_3_ powders	150	10	350	[34]
Mesoporous WO_3_ thin film	100	3	>200	[35]
MoO_3_ lameller	180 to 300	10	118	[36]
CdO nanowire (porous)	100	150	>150	[37]
SnO_2_-core/ZnO-shell nanofibers	300	70 to 2,000	20 to 320	[38]
ZnGa_2_O_4_-core/ZnO-shell nanowires	250	1	260	[39]

**Figure 4:**
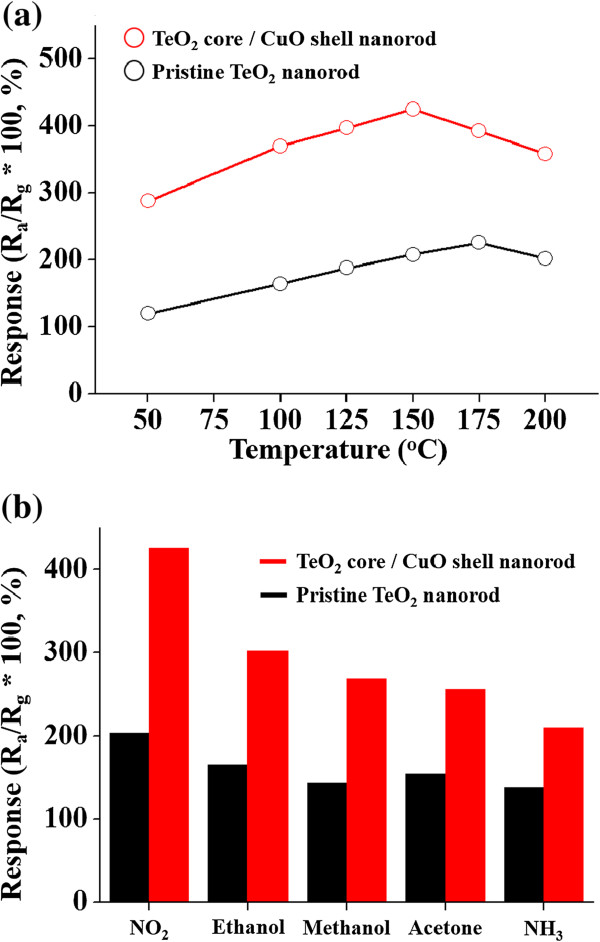
**Responses of the pristine TeO**_**2 **_**nanorod and TeO**_**2**_**/CuO core-shell nanorod gas sensors. (a)** Responses of the pristine TeO_2_ nanorod and TeO_2_/CuO core-shell nanorod gas sensors to NO_2_ as a function of the operation temperature. **(b)** Responses of the pristine TeO_2_ nanorod and TeO_2_/CuO core-shell nanorod gas sensors to different gases.

Figure 4a shows the responses of the pristine TeO_2_ nanorod and TeO_2_/CuO core-shell nanorod sensors to NO_2_ gas as a function of the operating time. The optimum operation temperature of TeO_2_/CuO core-shell nanorod sensor was 150°C, whereas that of the pristine TeO_2_ nanorod sensor was 175°C. This result reveals that encapsulation of TeO_2_ nanorods with a CuO thin film resulted in a 25°C decrease in operation temperature. Figure 4b exhibits the selectivity of the pristine and Bi_2_O_3_ nanoparticle-decorated In_2_O_3_ nanorod sensors to NO_2_ gas over other gases. The sensors showed the highest response to ethanol among different gases at the same concentration of 200 ppm at 150°C.

**Figure 5:**
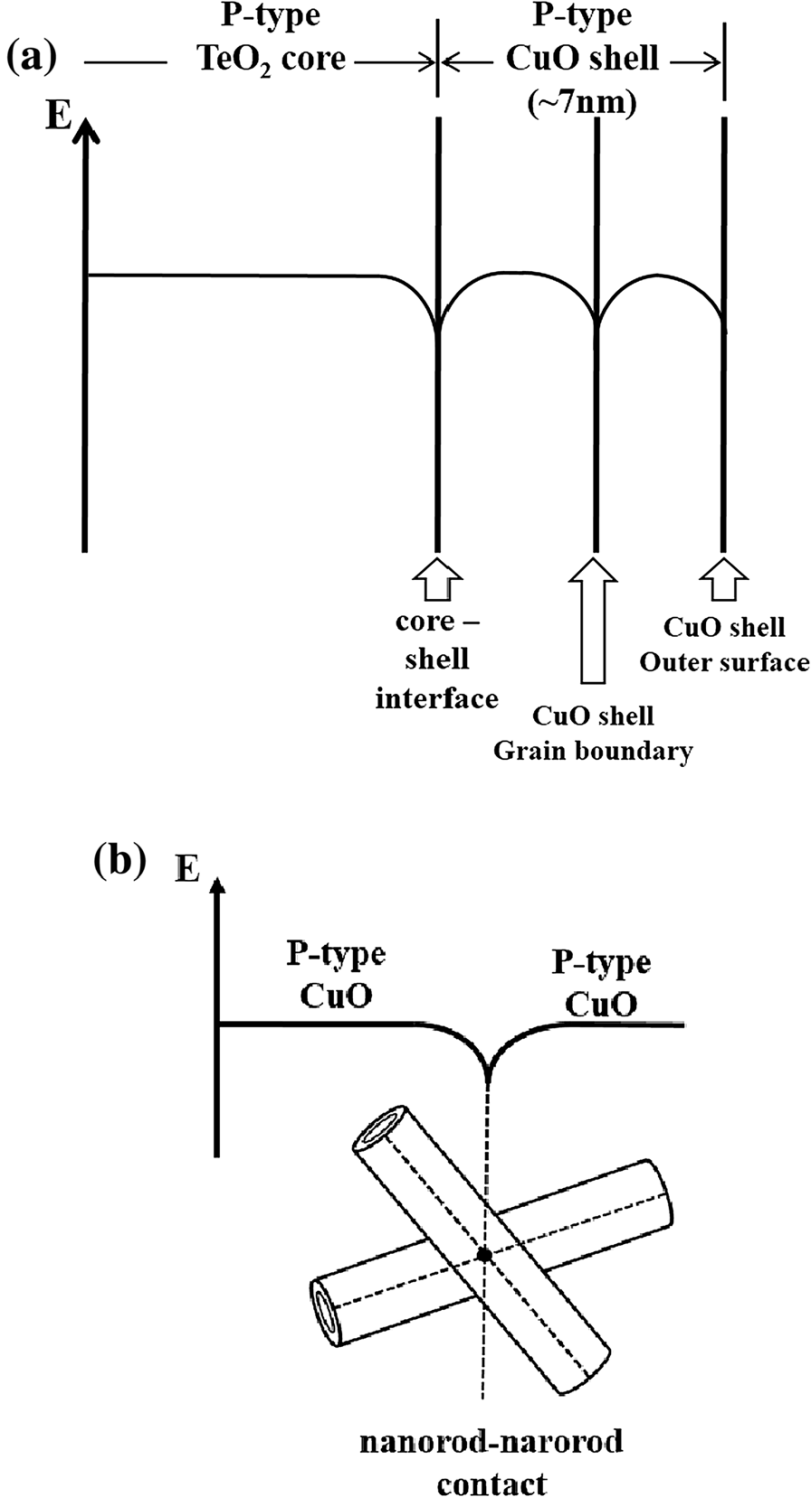
**Schematic energy diagram showing three different potential barriers.** Schematic energy diagram showing three different potential barriers formed in the multiple networked TeO_2_/CuO core-shell nanorod sensor: **(a)** one at a TeO_2_/CuO core-shell interface and another at a polycrystalline CuO shell grain boundary and **(b)** the third at a nanorod-nanorod contact.

The underlying mechanism of the enhanced TeO_2_/CuO core-shell nanorods can be explained using a barrier-controlled carrier transport mechanism [9,10]. Potential barriers form at three places in the multiple networked TeO_2_/CuO core-shell nanorod sensor: at the core-shell interface, the shell grain boundary [40], and the nanorod-nanorod contact. First, the potential barrier at core-shell interface is due to the high density of interface states in the TeO_2_-CuO interfacial region. The carriers near the interface are trapped by interface states, so that a depletion layer forms over the TeO_2_ core region near the interface to the CuO shell region near the interface. In addition to depletion layer formation, a potential barrier is created at the core-shell interface due to the carrier trapping as shown in Figure 5a [41]. The potential barrier is drawn in the negative energy direction, i.e. the downward direction in Figure 5a because the carriers trapped in the interface are mostly holes residing in p-type TeO_2_ core and the p-type CuO shell in the vicinity of the core-shell interface. The other two potential barriers that should be overcome by carriers on their pathways before they reach the electrode of the sensor are at the CuO-CuO homojunction, where two nanorods contact each other (Figure 5b) and at the grain boundary in the polycrystalline CuO shell layers (Figure 5a). The contributions of these two potential barriers might be smaller than that of the potential barrier at the TeO_2_-CuO interface because of much smaller numbers of grain boundaries and nanorod-nanorod contacts compared to that of the core-shell interfaces. Each nanorod has a core-shell interface, whereas a CuO shell contains a small number of grain boundaries because it is as thin as approximately 7 nm and the possibility of two nanorods contacting each other in a multiple networked nanorod sensor is generally quite low. Carrier transport is facilitated or restrained because of these energy barriers by adsorption and desorption of gas molecules, resulting in a larger change in resistance, i.e., an enhanced response of the core-shell nanorod sensor to NO_2_ gas. In other words, the heights of the potential barriers are modulated at the three places, resulting in enhanced response of the sensor to the gas.

## Conclusions

TeO_2_/CuO core-shell nanorods were synthesized using a two-step process: the synthesis of TeO_2_ nanorods by thermal evaporation of Te powder and sputter deposition of CuO. The cores and shells of the nanorods were single crystal TeO_2_ and polycrystalline CuO, respectively. The responses of the TeO_2_ nanorods to NO_2_ were improved approximately 2.1- to 2.1-fold at NO_2_ concentrations of 0.5 to 10 by coating them with CuO. The responses of the core-shell nanorods to NO_2_ gas were also comparable or superior to those of the other metal oxide semiconductor nanostructured sensors reported previously. The enhanced response of the TeO_2_/CuO core-shell nanorods to NO_2_ gas may be due to modulation of the heights of the potential barriers formed at three different places in the multiple networked 1D nanostructure sensor: the TeO_2_ core-CuO shell interface, the CuO-CuO homojunction at the contact of two core-shell nanorods, and the grain boundaries in the polycrystalline CuO shell layers.

## Competing interests

The authors declare that they have no competing interests.

## Authors’ contributions

All the authors contributed equally to the paper. All authors read and approved the final manuscript.
